# Low and high dose methamphetamine differentially regulate synaptic structural plasticity in cortex and hippocampus

**DOI:** 10.3389/fncel.2022.1003617

**Published:** 2022-11-02

**Authors:** Jiuyang Ding, Jian Huang, Xiang Tang, Lingyi Shen, Shanshan Hu, Jiaojiao He, Ting Liu, Zhixing Yu, Yubo Liu, Qiyan Wang, Jiawen Wang, Na Zhao, Xiaolan Qi, Jiang Huang

**Affiliations:** ^1^School of Forensic Medicine, Guizhou Medical University, Guiyang, China; ^2^Key Laboratory of Endemic and Ethnic Diseases, Ministry of Education, Guizhou Medical University, Guiyang, China; ^3^Guangzhou Key Laboratory of Forensic Multi-Omics for Precision Identification, School of Forensic Medicine, Southern Medical University, Guangzhou, China; ^4^Department of Children Rehabilitation, Children’s Hospital of Chongqing Medical University, Chongqing, China; ^5^Ministry of Education Key Laboratory of Child Development and Disorders, Children’s Hospital of Chongqing Medical University, Chongqing, China; ^6^National Clinical Research Center for Child Health and Disorders, Children’s Hospital of Chongqing Medical University, Chongqing, China; ^7^China International Science and Technology Cooperation Base of Child Development and Critical Disorders, Children’s Hospital of Chongqing Medical University, Chongqing, China; ^8^Chongqing Key Laboratory of Pediatrics, Children’s Hospital of Chongqing Medical University, Chongqing, China; ^9^School of Basic Medical Sciences, Guizhou Medical University, Guiyang, China; ^10^Good Clinical Practice Center, Affiliated Hospital of Zunyi Medical University, Zunyi, China; ^11^Key Laboratory of Environmental Pollution Monitoring and Disease Control, Ministry of Education, School of Public Health, Guizhou Medical University, Guiyang, China; ^12^State Key Laboratory of Functions and Applications of Medicinal Plants, Key Laboratory of Pharmaceutics of Guizhou Province, Guizhou Medical University, Guiyang, China

**Keywords:** synapse, structural plasticity, prefrontal cortex, hippocampus, methamphetamine

## Abstract

Psychostimulants, such as methamphetamine (METH) can induce structural remodeling of synapses by remodeling presynaptic and postsynaptic morphology. Escalating or long-lasting high dose METH accounts for neurodegeneration by targeting multiple neurotransmitters. However, the effects of low dose METH on synaptic structure and the modulation mechanism remain elusive. This study aims to assess the effects of low dose (2 mg/kg) and high dose (10 mg/kg) of METH on synaptic structure alternation in hippocampus and prefrontal cortex (PFC) and to reveal the underlying mechanism involved in the process. Low dose METH promoted spine formation, synaptic number increase, post-synaptic density length elongation, and memory function. High dose of METH induced synaptic degeneration, neuronal number loss and memory impairment. Moreover, high dose, but not low dose, of METH caused gliosis in PFC and hippocampus. Mechanism-wise, low dose METH inactivated ras-related C3 botulinum toxin substrate 1 (Rac1) and activated cell division control protein 42 homolog (Cdc42); whereas high dose METH inactivated Cdc42 and activated Rac1. We provided evidence that low and high doses of METH differentially regulate synaptic plasticity in cortex and hippocampus.

## Introduction

Methamphetamine (METH) has been one of the most widely abused drugs worldwide with estimated 27 million users (Ni et al., 2022). As a second-line treatment drug for attention deficit hyperactivity disorder and obesity, METH treatment is highly limited due to its neurotoxic effect (Chen et al., 2022). In addition to neurotoxicity, METH can lead to hepatotoxicity, aortic aneurysm, renal dysfunction, etc. (Ding et al., 2022; Luo et al., 2022; Zhang et al., 2022).

In central nervous system, METH functions as an enhancer for monoamine neurotransmitter releasing (Wang et al., 2022). Beside its neurotransmitter modulation effect in synapses, METH has been also proven to regulate synaptic structure (Yan et al., 2019). METH (2 mg/kg) significantly increases synaptic density of medium spiny neurons in nucleus accumbens (Tu et al., 2019). METH (2 mg/kg) also increases synaptic density and post synaptic density (PSD) thickness in hippocampus (Liang et al., 2022). In contrast, METH (30 mg/kg) induces striatal pre- and post-synaptic damages shown by dopamine terminal marker staining (Xu et al., 2005). The inconsistent results showed that the dosage difference of METH could differently regulate synaptic plasticity. However, the morphological evidences of synaptic plasticity and the specific mechanisms related to different METH dosages on regulation of synaptic structure remain unknown.

It is now widely accepted that METH differentially regulates glial function in different brain regions. The escalating dose of METH (1–5 mg/kg) induces glial activation in cortex and hippocampus (Ding et al., 2020a). Moreover, the activated responses from microglia and astrocytes result in an enhanced synaptic pruning, leading to pathological synaptic plasticity (Lall et al., 2021). This data suggested that the METH induced glial function might be involved in synaptic structure alteration.

The Rho family of small guanosine triphosphatases (GTPases) has been shown to be related to dendritic remodeling. Recent studies on Rac1 and Cdc42, both are members of Rho GTPase, may differentially regulate dendritic morphology in nucleus accumbens (Tu et al., 2019). Rac1 was activated in a cocaine mice model, leading to dendritic structural plasticity in caudate and putamen (Li et al., 2015). However, the influence on Rac1 and Cdc42 activity with different doses of METH on a mice model has not been studied.

To investigate the effect of different doses of METH on synaptic structures, we used 2 mg/kg as low dose, and 10 mg/kg as high dose, respectively. We found different doses of METH differentially regulates Rac1 and Cdc42 activity and leads to different synaptic structure plasticity. Our study reveals an important role of Rho GTPase in regulating METH induced synaptic plasticity in prefrontal cortex and hippocampus.

## Materials and methods

### Animals

C57BL/6J mice (male 22–26 g, 4–8 weeks old) were purchased from the Laboratory Animal Center of Guizhou Medical University (Guizhou, China). All mice were kept (4 mice per cage) under a controlled environment under a 12 h light-dark cycle with *ad libitum* access to food and water. All animal experiments were preapproved by the Institutional Animal Care and Use Committee of Guizhou Medical University and were performed according to the National Institutes of Health guide.

### Methamphetamine exposure and experimental groups

**FIGURE 1:**
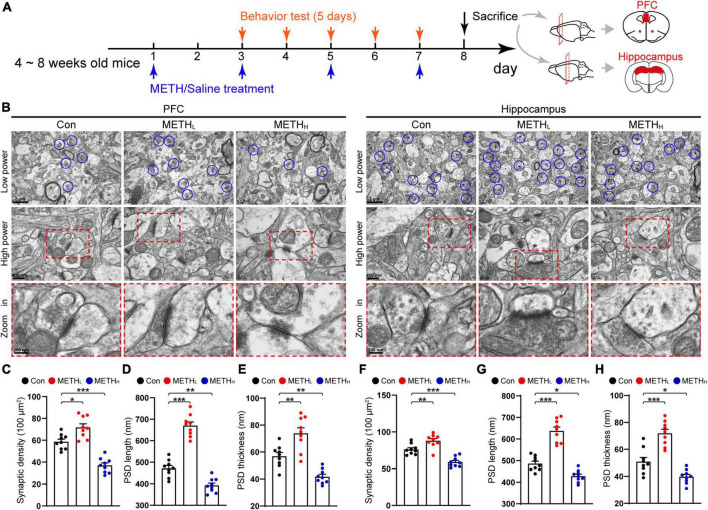
Low and high dose methamphetamine (METH) modulate ultrastructure and density of synapse in cortex and hippocampus. **(A)** Experimental protocol. **(B)** Representative electron micrographs of synapses in cortex and hippocampus of Con, METH_L_ and METH_H_ mice groups. Blue circles indicate synapses. **(C)** Quantification of synaptic density in cortex. One-way ANOVA, *F* (2, 26) = 43.964, *p* < 0.001. **(D)** Quantification of PSD length in cortex. One-way ANOVA, *F* (2, 26) = 105.581, *p* < 0.001. **(E)** Quantification of PSD thickness in cortex. One-way ANOVA, *F* (2, 26) = 29.393, *p* < 0.001. **(F)** Quantification of synaptic density in hippocampus. One-way ANOVA, *F* (2, 26) = 32.33, *p* < 0.001. **(G)** Quantification of PSD length in hippocampus. One-way ANOVA, *F* (2, 26) = 68.342, *p* < 0.001. **(H)** Quantification of PSD thickness in hippocampus. One-way ANOVA, *F* (2, 26) = 37.364, *p* < 0.001. *n* = 9 slices, 3 slices per mouse. *p < 0.05, **p < 0.01, ***p < 0.001 by one-way ANOVA and Dunnett’s *post hoc* analysis.

Methamphetamine (METH) administration (purity > 99%, National Institutes for Food and Drug Control, Guangzhou, China) was arranged according to Figure 1A. The doses of METH were set at 2 mg/kg as the low dose and 10 mg/kg as high dose, respectively according to the previous studies (Wen et al., 2019; Fan et al., 2020; Wang et al., 2021). The METH was dissolved in 0.9% saline at the concentration of 4 mg/ml. The volume of METH solution was calculated as [(Body weight/dose) × 4 mg/ml]. The mice were divided into three groups as follows:

Con: Control group. Saline was administered intraperitoneally in place of METH;

METH_*L*_: Low dose METH mice group. METH (2 mg/kg body weight) was injected intraperitoneally once daily on day 1, 3, 5, and 7;

METH_*H*_: High dose METH mice group. METH (10 mg/kg body weight) was injected intraperitoneally once daily on day 1, 3, 5, and 7;

### Spine morphology imaging analysis

We used intracellular injection of Lucifer Yellow dye to visualize the spine morphology as described before (Ding J. et al., 2021). Briefly, the mice brain tissues were acquired and fixed in 4% PFA for 4 hrs. Tissue sections (150μm in thickness) were made using a microtome (VT 1200, Leica, Germany). A micropipete was used to inject 4% Lucifer Yellow dye (L0259, Sigma-Aldrich, Germany) into the neurons in cortex and hippocampus. Then the fluorescence of the neurons was visualized with a confocal microscope (LSM880, Carl Zeiss, Germany). Three mice per group and 3 neurons per mouse were used in the experiment.

### Transmission electron microscope analysis

The TEM samples of mice brain tissues were performed as described in our previous studies (Ding et al., 2020a). Briefly, brain tissues were fixed in 2.5% glutaraldehyde (Millipore Sigma, Burlington, MA, USA) at 4°C for 12 hrs, embedded in Epon resin (Polyscience, Inc. Eppelheim, Germany) at 65°C for 2 days. Then the tissues were sectioned (7 nm in thickness) using an ultramicrotome (EM UC7, Leica, Wetzlar, Germany). Ultrathin sections were stained with lead citrate before subjected for imaging by an electron microscope (Tecnai G2, FEI, CA, USA) equipped with a 4k CCD camera for image capture. Three mice per group and 3 serial sections per mouse were conducted in the experiment.

### Western blot

Brain tissues were acquired and stored in a liquid nitrogen tank. Hippocampal and cortical tissues were homogenized in lysis buffer. For GTP bound Rac1 and Cdc42, lysates were incubated with GST-tagged PAK (p21-activated kinase)-PBD agarose bead (Cytoskeleton, PAK02). Beads were washed with lysis buffer before subjecting to SDS-PAGE. Target proteins were transferred onto nitrocellulose membranes, which were incubated with Rac1 (610650, 1:1,000 dilution, BD Transduction Laboratories, USA) and Cdc42 (610929, 1:1000 dilution, BD Transduction Laboratories, USA) overnight at 4°C. HRP-conjugated secondary antibodies were incubated with the membranes for 1 hrs. Intensities of detected protein bands were analyzed using Image J. The total Rac1 and Cdc42 were tested using a routine immunoblot procedure. The β-actin (ab8226, 1:2000 dilution, Abcam, USA) was used as an internal control.

### Nissl staining and immunohistochemistry staining

Mice brain tissues containing cortex and hippocampus were collected before being fixed in 4% PFA for 12 h. The samples were dehydrated and then embedded in wax. A microtome (RM2235, Leica, Germany) was used to acquire 5 μm sections. For IHC staining, the sections were incubated with Anti-Synaptophysin antibody (ab32127, 1:200 dilution, Abcam, USA), Anti-SNAP25 antibody (ab109105, 1:200 dilution, Abcam, USA), Anti-PSD95 antibody (ab18258, 1:200 dilution, Abcam, USA) before antigen recovery. The three targeted proteins were visualized using 3, 3 – diaminobenzidine (DAB) kits (CW2069, CWBio, China). Images were obtained using a microscope (CX23, Olympus, Japan). Three mice per group and 3 serial sections per mouse were conducted in the experiment.

Nissl staining was performed according to the manufacturer’s protocols of the staining kit (G1434, Solarbio life science, Beijing, China). Briefly, the 5 μm brain sections were stained with methylene blue solution for 10 min before placed in Nissl differentiation solution for 3 sec. Sections were dehydrated in 100% alcohol. Images were acquired using a microscope (CX23, Olympus, Japan). Three mice per group and 3 serial sections per mouse were conducted in the experiment.

### Morris water maze test

Mice were conducted MWM test from 3th day to 7th day. Briefly, the circular water tank (120 cm in diameter, 40 cm in height) was filled with opaque water. A movable platform was submerged (2 cm below the water surface) in one of the four quadrants. Mice were placed in the water to find the platform within 60 sec and to remain on it for at least 5 seconds. Mice were allowed to stay on the platform for 20 sec if they did not find the platform within 60 sec. The escape latency was recorded. On day 7, the platform was removed for conducting probe trial. The time spent in the quadrant where the platform was previously located was recorded.

### Statistical analysis

All analyses were performed by a blinded researcher. All data were expressed as the mean (M) ± standard error of mean (SEM). Statistical analysis was conducted using one-way ANOVA or two-way repeated measures ANOVA followed by Bonferroni’s, LSD or Dunnett’s multiple comparisons test using SPSS version 22.0 (IBM, New York, USA) and GraphPad 8 (GraphPad, USA). The significance was set at the level of *p* < 0.05. Statistical parameters, including *F* and *p* values, and analytical method used for each analysis are listed in the figure legends.

## Results

### Low dose and high dose methamphetamine differentially modulates ultrastructure of synapses

To assess the impact of METH on the synaptic ultrastructure in cortex and hippocampus, we imaged synaptic morphology using TEM (Figure 1B). Low dose METH mice showed an increased synaptic density, a longer PSD length, a thicker PSD thickness than control mice. While high dose METH mice exhibited synaptic degeneration including a decreased synaptic density, a shorter PSD length and a thinner PSD thickness compared with the control mice both in cortical and hippocampal areas (Figures 1C–H).

### Effect of different doses methamphetamine on structural plasticity of spines

**FIGURE 2:**
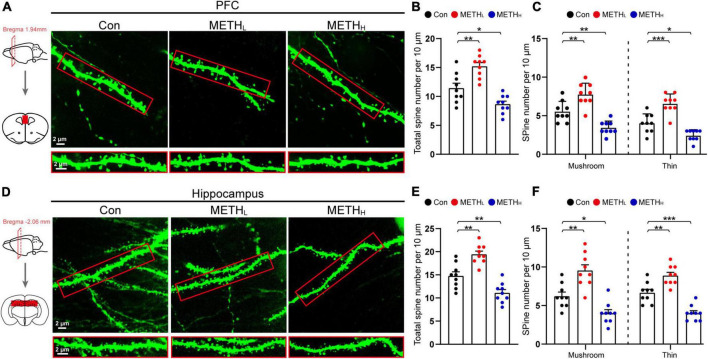
Effect of different doses of methamphetamine on structural plasticity of spines. **(A)** Representative images of spines by Lucifer Yellow intra-neuronal injection in cortex. The red squares were the areas where the Lucifer Yellow dye was injected. **(B)** Quantification of total spine number in cortex. One-way ANOVA, *F* (2, 26) = 22.978, *p* < 0.001. **(C)** Quantification of mushroom-type and thin-type spine numbers in cortex. One-way ANOVA, mushroom: *F* (2, 26) = 22.69, *p* < 0.001, thin: *F* (2, 26) = 32.719, *p* < 0.001. **(D)** Representative images of spines by Lucifer Yellow intra-neuronal injection in hippocampus. **(E)** Quantification of total spine number in hippocampus. One-way ANOVA, *F* (2, 26) = 29.747, *p* < 0.001. **(F)** Quantification of mushroom-type and thin-type spine number in hippocampus. One-way ANOVA, mushroom: *F* (2, 26) = 22.892, *p* < 0.001, thin: *F* (2, 26) = 37.096, *p* < 0.001. *n* = 9 neurons, 3 neurons per mouse. *p < 0.05, **p < 0.01, ***p < 0.001 by one-way ANOVA and Dunnett’s *post hoc* analysis.

Next, we conducted Lucifer Yellow dye injection which could show the post-synaptic dendritic morphology (Figures 2A,D). The total spine number in METH_*L*_ mice was higher than that of the control. However, the number of spines was significantly reduced in METH_*H*_ mice compared with control mice (Figures 2B,E). We further conducted spine type classification according to the shape of the spines. We found that the number of mushroom-type and thin type spines increased in low dose METH treated mice comparing with the control group. However, the number of thin-type and mushroom-type spines were lower in METH_*H*_ mice than those in control mice both in cortical and hippocampal areas (Figures 2C,F).

### Effect of low dose and high dose methamphetamine on synaptic proteins expression

**FIGURE 3:**
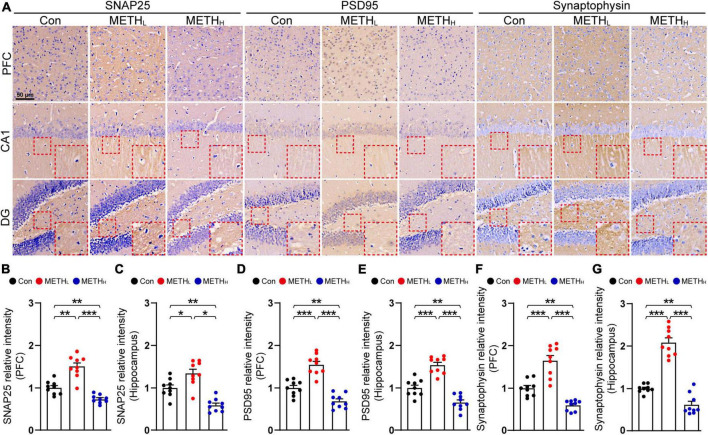
Effect of low dose and high dose methamphetamine (METH) on synaptic protein levels. **(A)** Representative IHC images of synaptic proteins SNAP25, PSD95 and Synaptophysin. **(B)** Relative intensity of SNAP25 level in Con, METH_L_ and METH_H_ mice cortex. One-way ANOVA, *F* (2, 26) = 40.098, *p* < 0.001. **(C)** Relative intensity of SNAP25 level in Con, METH_L_ and METH_H_ mice hippocampus. One-way ANOVA, *F* (2, 26) = 24.861, *p* < 0.001. **(D)** Relative intensity of PSD95 level in Con, METH_L_ and METH_H_ mice cortex. One-way ANOVA, *F* (2, 26) = 43.68, *p* < 0.001. **(E)** Relative intensity of PSD95 level in Con, METH_L_ and METH_H_ mice hippocampus. One-way ANOVA, *F* (2, 26) = 48.631, *p* < 0.001. **(F)** Relative intensity of Synaptophsyin level in Con, METH_L_ and METH_H_ mice cortex. One-way ANOVA, *F* (2, 26) = 42.155, *p* < 0.001. **(G)** Relative intensity of Synaptophsyin level in Con, METH_L_ and METH_H_ mice hippocampus. One-way ANOVA, *F* (2, 26) = 88.27, *p* < 0.001. *n* = 9 sections, 3 sections per mouse. *p < 0.05, **p < 0.01, ***p < 0.001 by one-way ANOVA and Bonferroni’s *post hoc* analysis.

Based on the synaptic morphology changes influenced by METH, we next assessed the synaptic proteins including SNPA25, PSD95 and Synaptophysin in PFC and hippocampus (Figure 3A). We observed increased levels of SNAP25, PSD95 and Synaptophysin in PFC and hippocampus after low dose METH exposure. In contrast, high dose METH exposure exhibited a decreased SNAP25, PSD95 and Synaptophysin protein levels than control mice (Figures 3B–G).

### High dose but not low dose of methamphetamine induced glial activation

**FIGURE 4:**
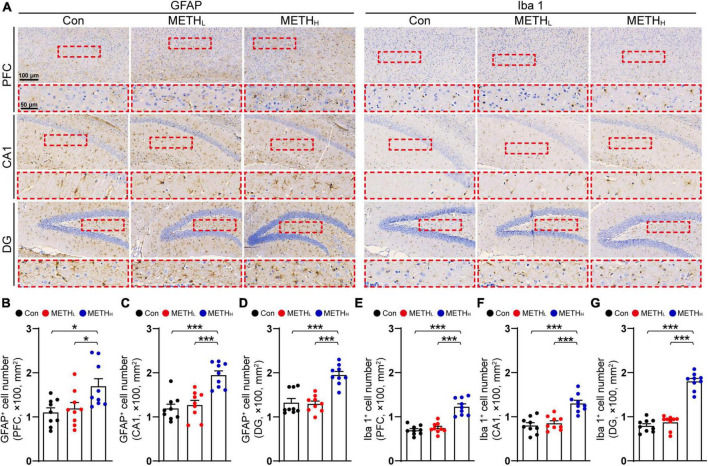
Effect of low dose and high dose methamphetamine (METH) on glial cell number in cortex and hippocampus. **(A)** Representative images of GFAP and Iba 1 IHC staining in cortex and hippocampus. **(B)** Quantification of GFAP^+^ cell density in cortex of control, METH_L_ and METH_H_ mice. One-way ANOVA, F (2, 26) = 5.005, *p* < 0.05. **(C)** Quantification of GFAP^+^ cell density in CA1 of control, METH_L_ and METH_H_ mice. One-way ANOVA, F (2, 26) = 18.17, *p* < 0.001. **(D)** Quantification of GFAP^+^ cell density in DG of control, METH_L_ and METH_H_ mice. One-way ANOVA, F (2, 26) = 23.114, *p* < 0.001. **(E)** Quantification of Iba 1^+^ cell density in cortex of control, METH_L_ and METH_H_ mice. One-way ANOVA, F (2, 26) = 38.623, *p* < 0.001. **(F)** Quantification of Iba 1^+^ cell density in CA1 of control, METH_L_ and METH_H_ mice. One-way ANOVA, *F* (2, 26) = 20.342, *p* < 0.001. **(G)** Quantification of Iba 1^+^ cell density in DG of control, METH_L_ and METH_H_ mice. One-way ANOVA, *F* (2, 26) = 103.087, *p* < 0.001. *n* = 9 sections, 3 sections per mouse. *p < 0.05, ***p < 0.001 by one-way ANOVA and Bonferroni’s *post hoc* analysis.

Glial cells participate in synaptic structure remodeling through pruning and phagocytosis. We questioned whether METH could affect glial activation and lead to synaptic degeneration. GFAP and Iba 1 IHC were conducted to analysis the number and morphology of astrocyte and microglia in cortex and hippocampus (Figure 4A). High dose METH caused an appreciably increase of astrocyte and microglia numbers compared with that of control mice. In METH treated mice, the number of glial cell processes is higher and they appeared longer, compared with that in control mice. However, the number of glial cells was not comparable between the low dose METH mice and the control group (Figures 4B–G).

### High dose but not low dose methamphetamine induced neuronal loss

**FIGURE 5:**
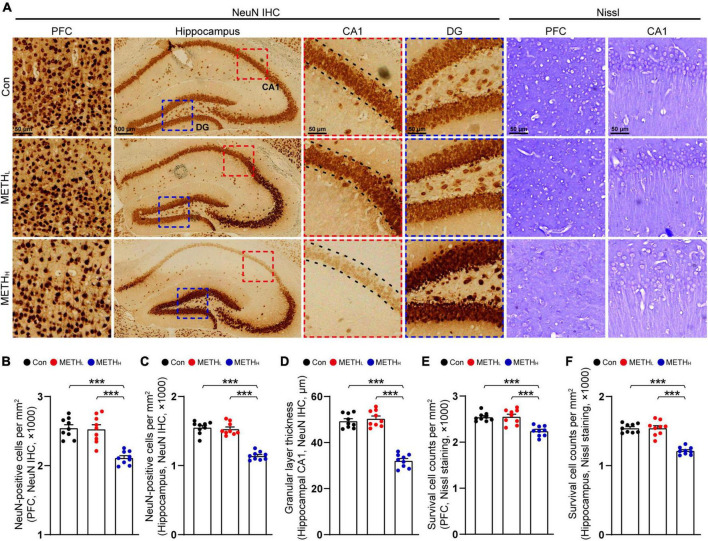
Effect of METH on neuronal number. **(A)** Representative figures of NeuN IHC staining and Nissl staining in cortex and hippocampus. **(B)** Quantification of NeuN^+^ cell density in cortex of control, METH_L_ and METH_H_ mice. One-way ANOVA, F (2, 26) = 23.786, *p* < 0.001. **(C)** Quantification of NeuN^+^ cell density in hippocampal area of control, METH_L_ and METH_H_ mice. One-way ANOVA, F (2, 26) = 72.334, *p* < 0.001. **(D)** Quantification of granular layer thickness in hippocampal CA1 area from NeuN IHC staining. One-way ANOVA, F (2, 26) = 95.847, *p* < 0.001. **(E)** Quantification of survival cell counts in cortex from Nissl staining. One-way ANOVA, F (2, 26) = 18.671, *p* < 0.001. **(F)** Quantification of survival cell counts in hippocampus from Nissl staining. One-way ANOVA, F (2, 26) = 53.05, *p* < 0.001. *n* = 9 sections, 3 sections per mouse. ***p < 0.001 by one-way ANOVA and Bonferroni’s *post hoc* analysis.

Next, we used NeuN IHC and Nissl staining to observe the neuronal number in cortex and hippocampus (Figure 5A). We found that the numbers of NeuN positive cells in control mice and METH_*L*_ mice were comparable. By contrast, high dose METH induced a reduction of neuronal number in cortex and hippocampus compared with saline treated mice (Figures 5B,C,E,F). The thickness of CA1 pyramidal cell layer in high dose METH mice was thinner comparing with that in control mice. No such changes found when comparing control mice with METH_*L*_ mice (Figure 5D).

### Low dose and high dose methamphetamine differentially regulated Rac1 and Cdc42 signaling

**FIGURE 6:**
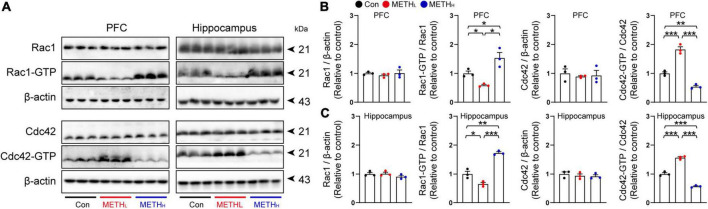
Methamphetamine (METH) regulated Rac1 and Cdc42 activity in hippocampus and cortex. **(A)** Representative immunoblots of Rac1, Rac1-GTP, Cdc42, Cdc42-GTP and β-actin in PFC and hippocampus. **(B)** Quantification of Rac1 and Cdc42 normalized to β-actin, Rac1-GTP normalized to Rac1, and Cdc42-GTP normalized to Cdc42 in PFC. One-way ANOVA, Rac1/β-actin, F (2, 8) = 0.393, *p* = 0.691, Rac1-GTP/Rac1, F (2, 26) = 15.933, *p* = 0.004, Cdc42/β-actin, F (2, 8) = 0.178, *p* = 0.841, Cdc42-GTP/Cdc42, F (2, 8) = 0.178, *p* < 0.001. **(C)** Quantification of Rac1 and Cdc42 normalized to β-actin, Rac1-GTP normalized to Rac1, and Cdc42-GTP normalized to Cdc42 in hippocampus. One-way ANOVA, Rac1/β-actin, F (2, 8) = 1.439, *p* = 0.309, Rac1-GTP/Rac1, F (2, 8) = 68.719, *p* < 0.001, Cdc42/β-actin, F (2, 8) = 0.451, *p* = 0.657, Cdc42-GTP/Cdc42, F (2, 8) = 209.847, *p* < 0.001. *n* = 3. *p < 0.05, **p < 0.01, ***p < 0.001 by one-way ANOVA and Bonferroni’s *post hoc* analysis.

To determine whether Rac1 and Cdc42 signaling were regulated by METH, we tested the Rac1 and Cdc42 activities by detecting their expression levels in cortex and hippocampus using immunoblot. Results showed that Rac1 activity was decreased, whereas Cdc42 activity was increased both in PFC and hippocampus after low METH treatment. High dose METH increased Rac1 activity and reduced Cdc42 activity both in PFC and hippocampus (Figure 6).

### Effects of low dose and high dose methamphetamine on mice spatial memory

**FIGURE 7:**
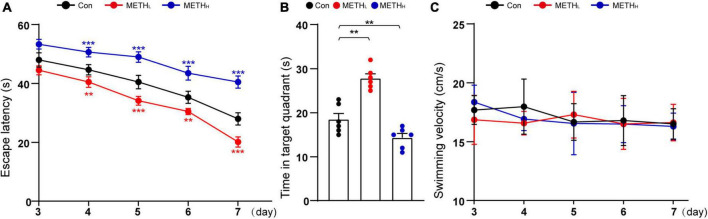
Effect of low dose and high dose methamphetamine (METH) on spatial memory. **(A)** Escape latency in MWM test. Two – way repeated measures ANOVA, day: *F* (4, 60) = 281.836, *p* < 0.001, treatment: *F* (2, 15) = 424.790, *p* < 0.001, day × treatment: *F* (8, 60) = 8.727, *p* < 0.001, 3th day: *F* (2, 26) = 32.477, *p* < 0.001, 4th day: *F* (2, 26) = 53.226, *p* < 0.001, 5th day: *F* (2, 26) = 95.271, *p* < 0.001, 6th day: *F* (2, 26) = 71.518, *p* < 0.001, 7th day: *F* (2, 26) = 162.271, *p* < 0.001. **(B)** Time in quadrant of different group mice. One-way ANOVA, F (2, 26) = 39.562, *p* < 0.001. **(C)** Swimming velocity in MWM test. Two – way repeated measures ANOVA, day: *F* (4, 60) = 1.206, *p* = 0.318, treatment: *F* (2, 15) = 0.462, *p* = 0.639, day × treatment: *F* (8, 60) = 0.528, *p* = 0.830, 3th day: *F* (2, 26) = 1.297, *p* = 0.302, 4th day: *F* (2, 26) = 1.29, *p* = 0.304, 5th day: *F* (2, 26) = 0.217, *p* = 0.808, 6th day: *F* (2, 26) = 0.044, *p* = 0.957, 7th day: *F* (2, 26) = 0.097, *p* < 0.908. *n* = 6. **p < 0.01, ***p < 0.001 by One – way ANOVA or Two – way repeated measures ANOVA and Bonferroni’s *post hoc* analysis.

We used MWM to assess the spatial memory of the mice treated with different doses of METH. Low dose METH treated mice, but not high dose ones, exhibited decreased escape latencies and longer time in target quadrant compared with control mice. However, high dose METH increased escape latencies and reduced the time in target quadrant compared with control mice (Figures 7A,B). Note that the swimming velocities of different group mice were comparable (Figure 7C).

## Discussion

In the present study, we showed high dose METH caused synaptic degeneration including a lower synaptic density, mature spine loss, and a compromised post-synaptic structure. High dose METH also induce neuronal loss and spatial memory impairment. Low dose METH could increase synaptic number, promote mature spine formation which account for memory function enhancement. Interestingly, we demonstrated that METH might modulate Rac1- and Cdc42- signaling pathway. In addition, high dose, but not low dose, METH might induce glial activation both in PFC and hippocampus.

Previous studies showed that METH, with 10 mg/kg treatment for 5 days, could induce neurodegeneration, whereas METH, with 2 mg/kg treatment for 4 days, improved memory function (Cao et al., 2018; Tu et al., 2019). Due to the discrepancies triggered by different doses of METH, we set 2 mg/kg and 10 mg/kg as our low dose and high dose treatment standards. Since synapse has been proposed as a neuronal substrate that influences many aspects of behaviors (Ru et al., 2021), we firstly analyzed the synaptic morphology through TEM. Interestingly, we found high dose METH caused synaptic degeneration, whereas low METH improved synaptic plasticity. These results echoed those studies showing that low dose METH (2 mg/kg) increased hippocampal synaptic number and the thickness of PSD, and high dose METH (10 mg/kg or 15 mg/kg) caused dopaminergic cell terminal degeneration (Ares-Santos et al., 2014; Liang et al., 2022).

To verify the synaptic density changes induced by two different doses of METH shown on TEM, we conducted spine type analysis using Lucifer Yellow fluorescence dye and intra-neuronal injection. After low dose METH exposure, the number of mature and immature spines, which could be identified as mushroom-type or thin-type, respectively, were increased. High dose METH triggered both mature and immature spine number loss. In line with this, changes in total number of spines were mainly driven by the alteration of mushroom-type and thin-type in METH mice model (Tu et al., 2019).

Based on the morphological analysis of the synapse, we conducted SNAP25, PSD95 and Synaptophysin IHC tests to determine whether synaptic proteins were modified by METH. An obvious increase of these synaptic proteins were observed in low dose METH mice in PFC and hippocampus whereas high dose METH induced a loss of these synaptic proteins. Some studies showed that Synaptophysin is increased in the entire hippocampus and is decreased in prefrontal cortex, and PSD95 is decreased in in the entire hippocampus and is increased in prefrontal cortex in 2 mg/kg METH mice using immunoblot (Fan et al., 2020). The possible explanation of our finding is that the protein detection method is IHC and the area of hippocampus we collected is from the dorsal part.

Previous studies have demonstrated that high dose METH induced neuronal loss in substantial nigra, hippocampus and cortex (Wen et al., 2019; Ding et al., 2020a). Our study revealed that high dose METH triggered cortical and hippocampal neuron loss. And the changes in neuronal number were mainly driven by the alteration of hippocampal CA1, but not DG. Interestingly, low dose of METH did not induce neuronal number alternation. We speculated that the neurotoxic effect of METH was dose dependent (Barr et al., 2006), and 2 mg/kg METH was non-toxic to the hippocampal and cortical neurons. Consistent with our results, recent studies found low dose METH (2 mg/kg, 8 consecutive days) increased the dendritic spine number in shell and core of ventral striatum. Moreover, they found mammalian target of the rapamycin (mTOR) was activated in nucleus accumbens of METH-sensitized mice. Inhibition of mTOR suppressed the METH induced spine number increasing (Huang et al., 2018).

Various studies showed that neuroinflammation and concomitant gliosis contribute to synaptic degeneration (Ding Z. et al., 2021), and large amount of METH could activate microglia and astrocyte, which lead to synaptic dysfunction (Canedo et al., 2021; Shi et al., 2022). In line with this view, we found that high dose METH triggered astrocyte and microglia activation. However, the low dose of METH was not able to induce gliosis. Since gliosis dependent synaptic pruning could lead to synapse loss (Lecca et al., 2022), we speculate that the high-dose-METH-induced glial activation might be responsible for the synaptic degeneration.

A number of previous studies reported that Rac1 activity is increased in neurodegenerative disease, such as Alzheimer’s disease (Borin et al., 2018; Wu et al., 2019). High dose METH might induce neurodegeneration in hippocampus and cortex (Ding et al., 2020b; Ru et al., 2022). Consistent with these findings, we found that Rac1 activity was increased in high dose METH treated mice. The Rac1 is known to activate multiple signaling pathways which could regulate synaptic remodeling through actin branching (Rouiller et al., 2008; Firat-Karalar and Welch, 2011). Moreover, Rac1 activation is associated with the decay of hippocampus-dependent spatial memory in mice and olfactory memory in flies (Wu et al., 2019), and learning and memory rely on synapse-specific modifications (Appelbaum et al., 2022). Rac1 inhibition rescued memory loss in AD mice (Wu et al., 2019). Thus, we thought that high dose METH might trigger Rac1 activation dependent synaptic loss, leading to an impaired spatial memory function. However, low dose of METH might inactivate Rac1, leading to synaptic plasticity and spine density increase. Our studies also revealed that Cdc42, another member of Rho GTPase, was activated in low dose and inactivated in high dose METH mice groups, respectively. To our knowledge, Cdc42 activation is responsible for spine formation and memory function enhancement. Mechanism-wise, Rac1 and Cdc42 were critical for METH induced synaptic plasticity. Previous studies showed that spine morphology is regulated by the activity of Rac1 and Cdc42, which control the actin formation. And the RhoA pathway modulates the spine morphology by influencing the balance between the Rac1 and Cdc42 activities (Martin-Camara et al., 2021).

**FIGURE 8:**
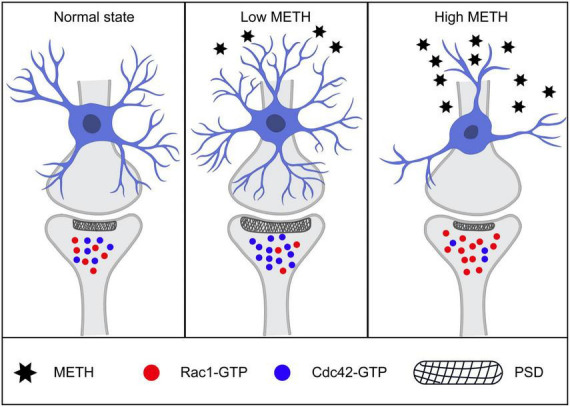
Schematic illustration of mechanisms of low and high methamphetamine (METH) modulated synaptic structure. Low dose of METH activated Cdc42 and inactivated Rac1, leading to spine formation, synaptic number increasing, post-synaptic density elongating and memory function enhancement. While high dose of METH induced Rac1-GTP increasing and Cdc42-GTP reducing, leading to synaptic degeneration, neuronal number loss and memory impairment.

In conclusion, low dose and high dose METH differentially regulated synaptic structural plasticity and Rac1 and Cdc42 signaling (Figure 8). Specifically, low dose METH inactivated Rac1 and activated Cdc42, leading to synaptic density increase and working memory function enhancement. High dose METH inactivated Cdc42 and activated Rac1, leading to synaptic degeneration and memory impairment. However, we did not test the downstream signaling pathways of Rac1 and Cdc42, which would be a subject of our future investigation. Our findings suggested that Rac1 and Cdc42 signaling may serve as a potential therapeutic strategy for METH induced synaptic plasticity.

## Data availability statement

The original contributions presented in this study are included in the article/supplementary material, further inquiries can be directed to the corresponding authors.

## Ethics statement

The animal study was reviewed and approved by Institutional Animal Care and Use Committee of Guizhou Medical University.

## Author contributions

JD and JH (14th author) conceived and designed the research. JD, JH (2nd author), XT, LS, SH, JH (6th author), ZY, YL, QW, JW, and NZ performed the experiments. TL and XQ: contributed reagents, materials, and analysis tools. JD and JH (2nd author) manuscript. All authors edited and approved the final version of the manuscript.
